# Supplementary material for: Cross-species analysis of LZTR1 loss-of-function mutants demonstrates dependency to RIT1 orthologs
Source: eLife. 2022 Apr 25;11:e76495. doi: 10.7554/eLife.76495 (PMC9068208; doi:10.7554/eLife.76495)
Supplement: Figure 1—source data 1. [file elife-76495-fig1-data1.zip › Figure 1 - source data/Figure 1 - source data 1.docx]

| Protein | Species | Uniprot | Identity (to human) |
| --- | --- | --- | --- |
| RIT1 | *Homo sapiens* | RIT1_HUMAN | 100% |
|  | *Pan troglodytes* | A0A2J8K4Z0_PANTR | 100% |
|  | *Sus scrofa* | Q06A93_PIG | 97.72% |
|  | *Mus musculus* | RIT1_MOUSE | 94.98% |
|  | *Gallus gallus* | H9L1B4_CHICK | 93.90% |
|  | *Xenopus laevis* | Q6AZU5_XENLA | 86.05% |
|  | *Danio rerio* | B3DJU3_DANRE | 75.83% |
|  | *Drosophila melanogaster* | Q7JMZ0_DROME | 55.50% |
|  | *Caenorhabditis elegans* | Not found | N/A |
|  | *Saccharomyces cerevisiae* | Not found | N/A |
| KRAS | *Homo sapiens* | RASK_HUMAN iso2B | 100% |
|  | *Pan troglodytes* | H2Q5M0_PANTR | 100% |
|  | *Sus scrofa* | A0A287B854_PIG | 99.47% |
|  | *Mus musculus* | RASK_MOUSE iso2B | 97.34% |
|  | *Gallus gallus* | A0A1D5NY37_CHICK | 98.94% |
|  | *Xenopus laevis* | RASK_XENLA | 97.33% |
|  | *Danio rerio* | Q6AZA4_DANRE | 96.28% |
|  | *Drosophila melanogaster* | RAS1_DROME | 79.79% |
|  | *Caenorhabditis elegans* | LET60_CAEEL | 79.23% |
|  | *Saccharomyces cerevisiae* | RAS1_YEAST  RAS2_YEAST | 55.85%  56.91% |
| LZTR1 | *Homo sapiens* | LZTR1_HUMAN | 100% |
|  | *Pan troglodytes* | H2QLA3_PANTR | 99.88% |
|  | *Sus scrofa* | A0A286ZYJ1_PIG | 95.48% |
|  | *Mus musculus* | LZTR1_MOUSE | 95.10% |
|  | *Gallus gallus* | A0A1D5P134_CHICK | 89.85% |
|  | *Xenopus laevis* | Q6NRT1_XENLA | 87.29% |
|  | *Danio rerio* | F1R533_DANRE | 87.61% |
|  | *Drosophila melanogaster* | LZTR1_DROME | 54.50% |
|  | *Caenorhabditis elegans* | Not found | N/A |
|  | *Saccharomyces cerevisiae* | Not found | N/A |
