# Supplementary figures and images for: Cross-species analysis of LZTR1 loss-of-function mutants demonstrates dependency to RIT1 orthologs
Source: eLife. 2022 Apr 25;11:e76495. doi: 10.7554/eLife.76495 (PMC9068208; doi:10.7554/eLife.76495)

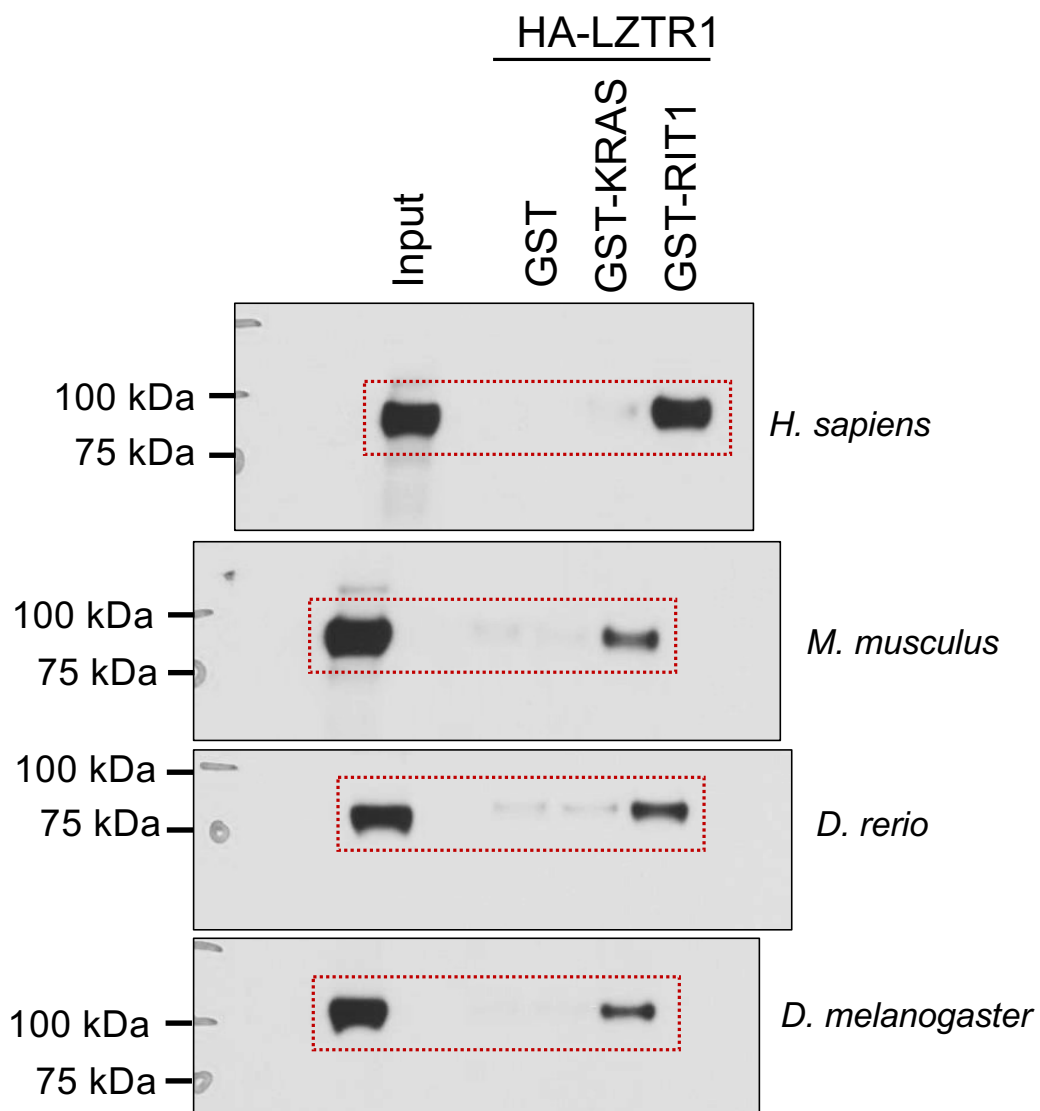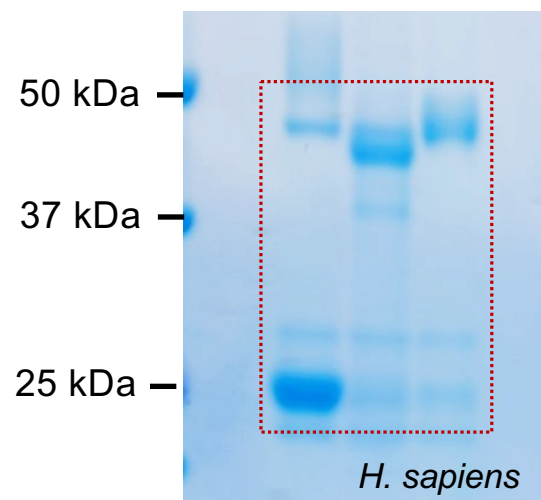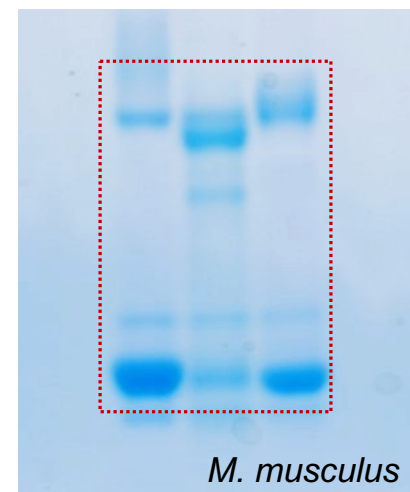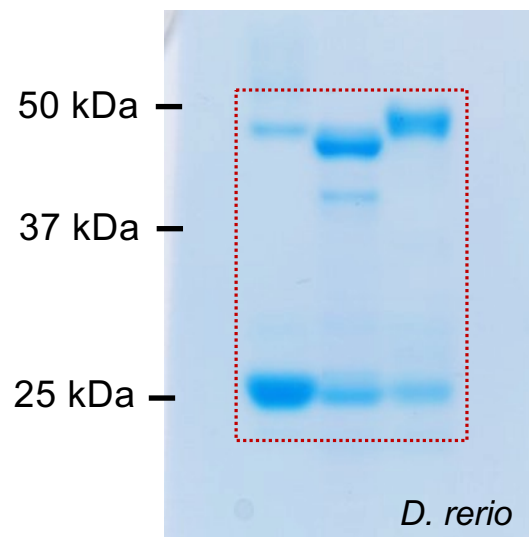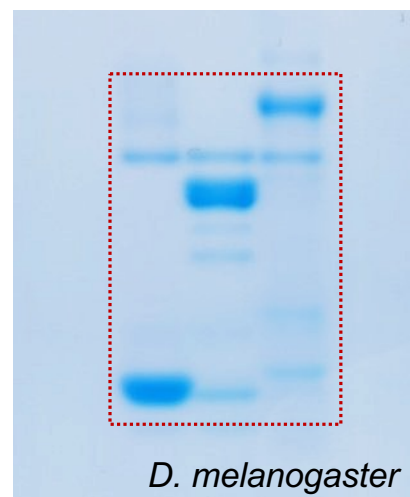

Supplement: Figure 1—source data 1. [file elife-76495-fig1-data1.zip › Figure 1 - source data/Figure 1 - source data 2.pdf]

|            |                         |                         |                          |                         |
|------------|-------------------------|-------------------------|--------------------------|-------------------------|
|            | <i>yw</i>               |                         | <i>Lztr1<sup>2</sup></i> |                         |
|            | <i>Ras<sup>HA</sup></i> | <i>Ric<sup>HA</sup></i> | <i>Ras<sup>HA</sup></i>  | <i>Ric<sup>HA</sup></i> |
| Transgene: | .                       | .                       | .                        | .                       |

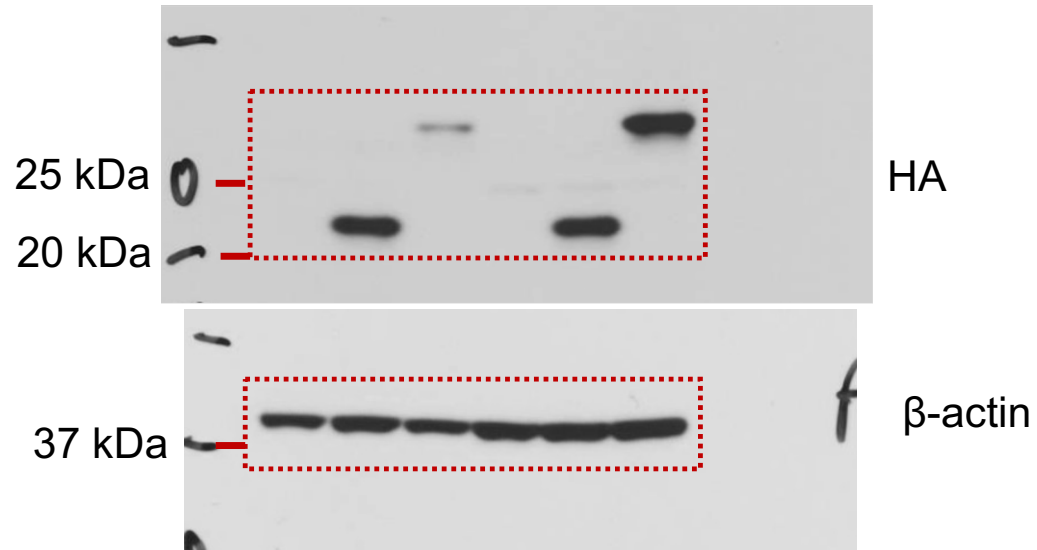

Supplement: Figure 2—source data 1. [file elife-76495-fig2-data1.zip › Figure 2 - source data/Figure 2 - source data 2.pdf]

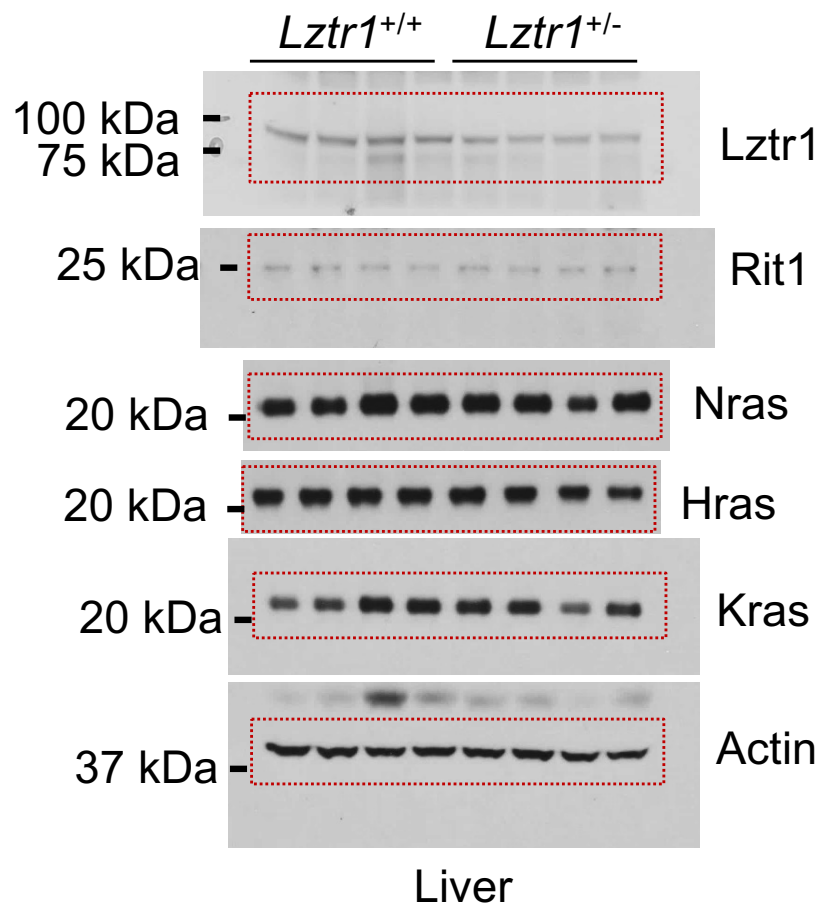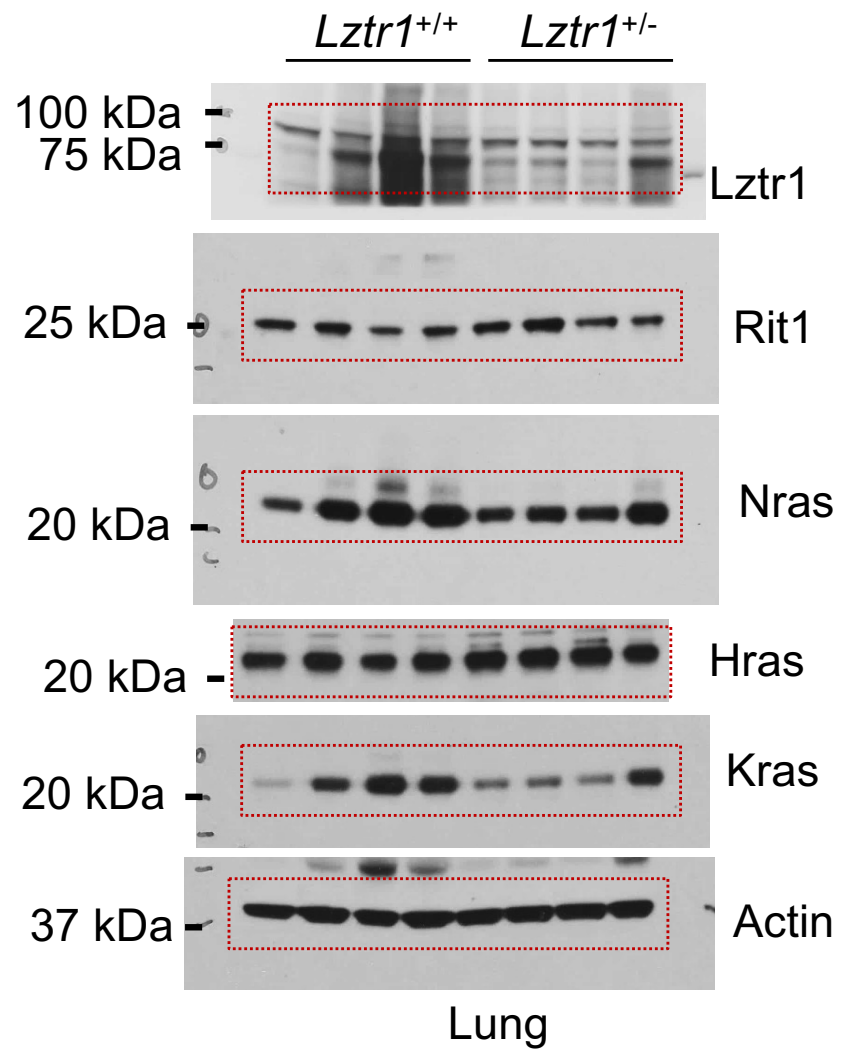

Supplement: Figure 3—source data 1. [file elife-76495-fig3-data1.zip › Figure 3 - source data/Figure 3 - supplement 1e - source data.pdf]

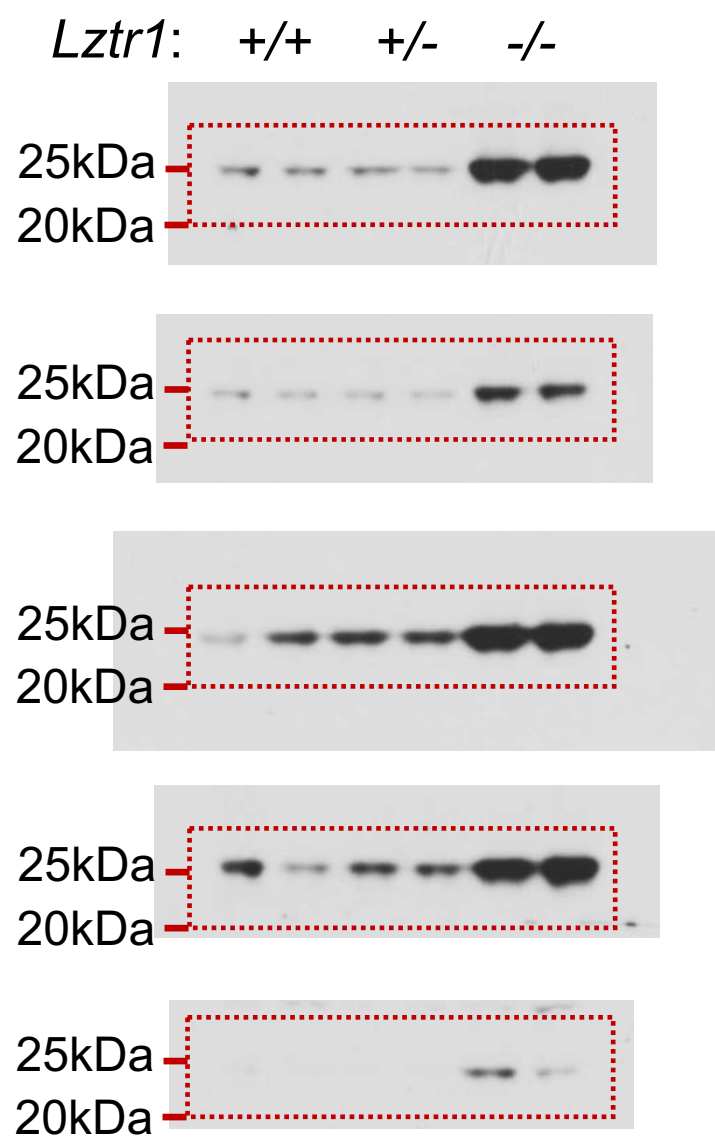

RIT1

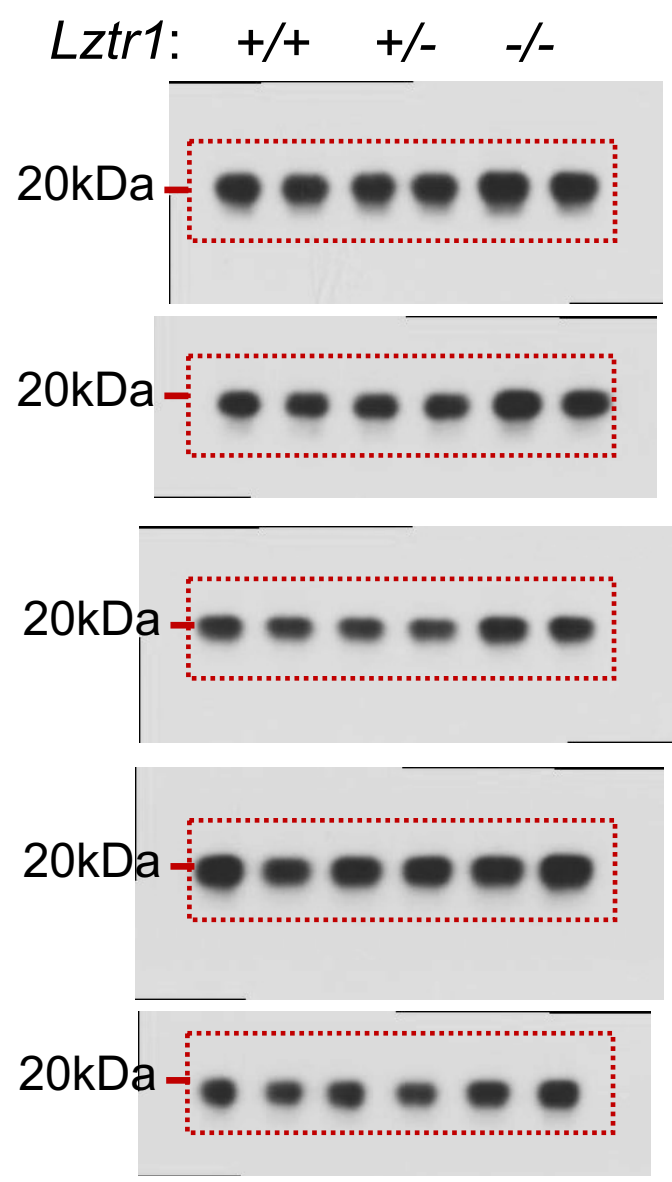

panRAS

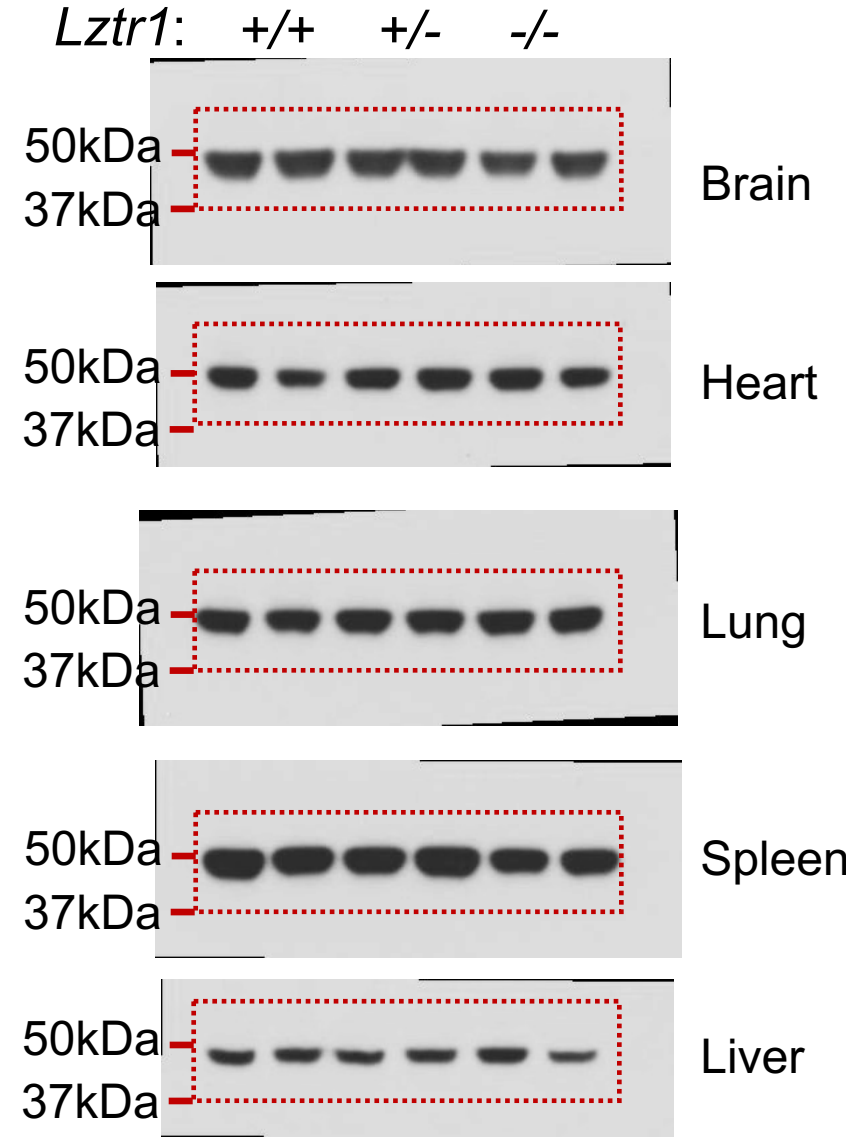

Tubulin

Supplement: Figure 3—source data 1. [file elife-76495-fig3-data1.zip › Figure 3 - source data/Figure 3 - source data 2.pdf]

10% FBS (h):      *Lztr1*<sup>+/+</sup>      *Lztr1*<sup>-/-</sup>      *Lztr1*<sup>+/+</sup>; *Rit1*<sup>-/-</sup>      *Lztr1*<sup>-/-</sup>; *Rit1*<sup>-/-</sup>  
                          0 15' 1 4 8 24    0 15' 1 4 8 24    0 15' 1 4 8 24    0 15' 1 4 8 24

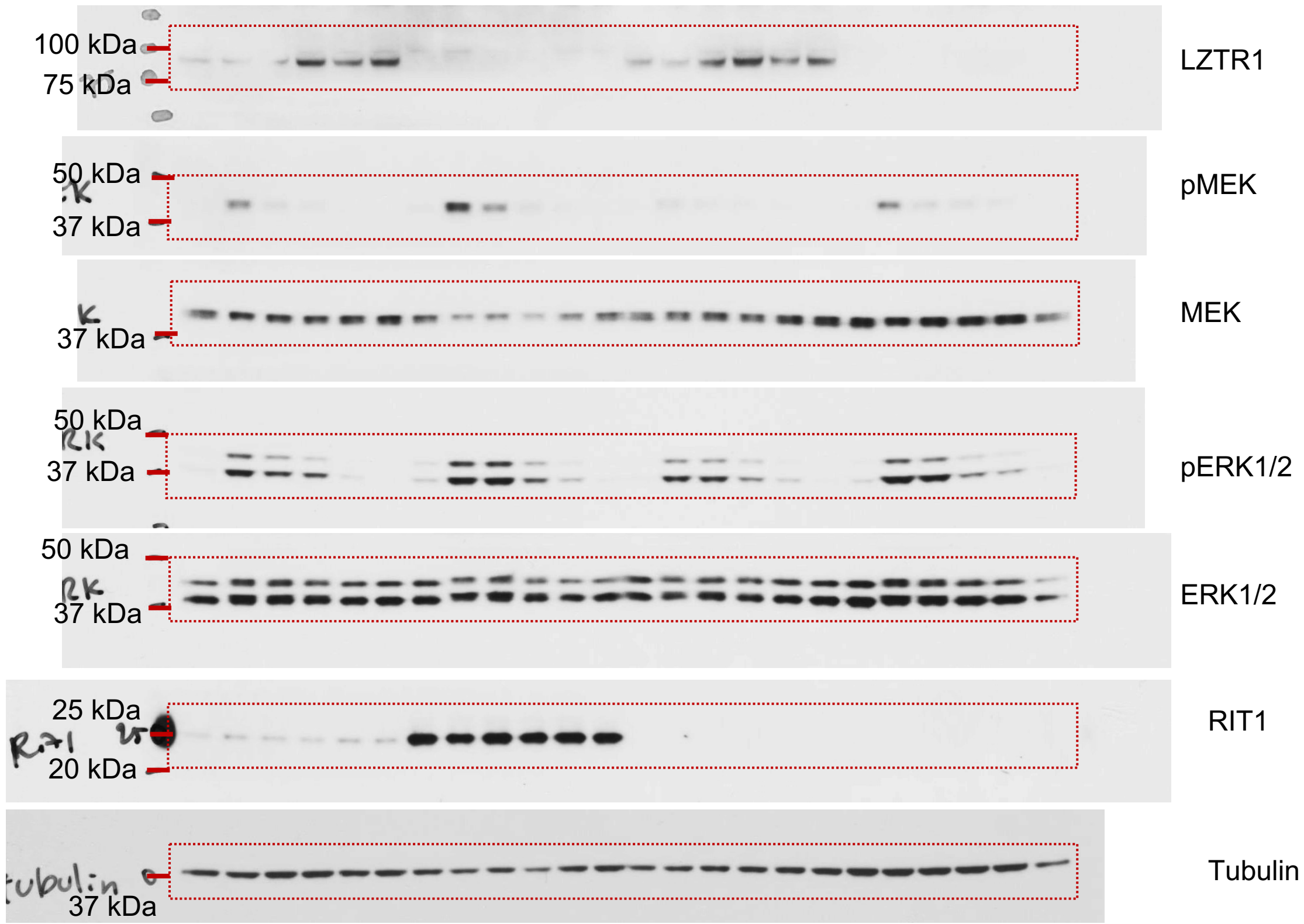

Supplement: Figure 4—source data 1. [file elife-76495-fig4-data1.zip › Figure 4 - source data/Figure 4 - source data 4.pdf]

*Lztr1*: +/+ -/- +/+ -/- -/-

*Rit1*: +/+ +/+ -/- -/- -/-

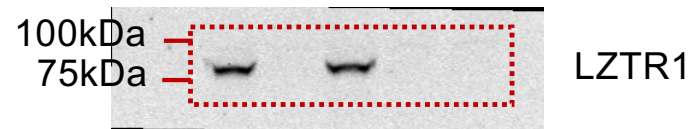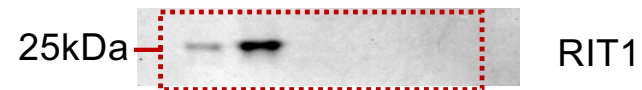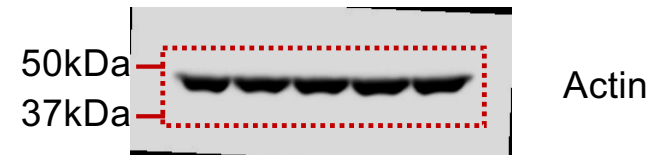

Supplement: Figure 4—source data 1. [file elife-76495-fig4-data1.zip › Figure 4 - source data/Figure 4 - source data 3.pdf]

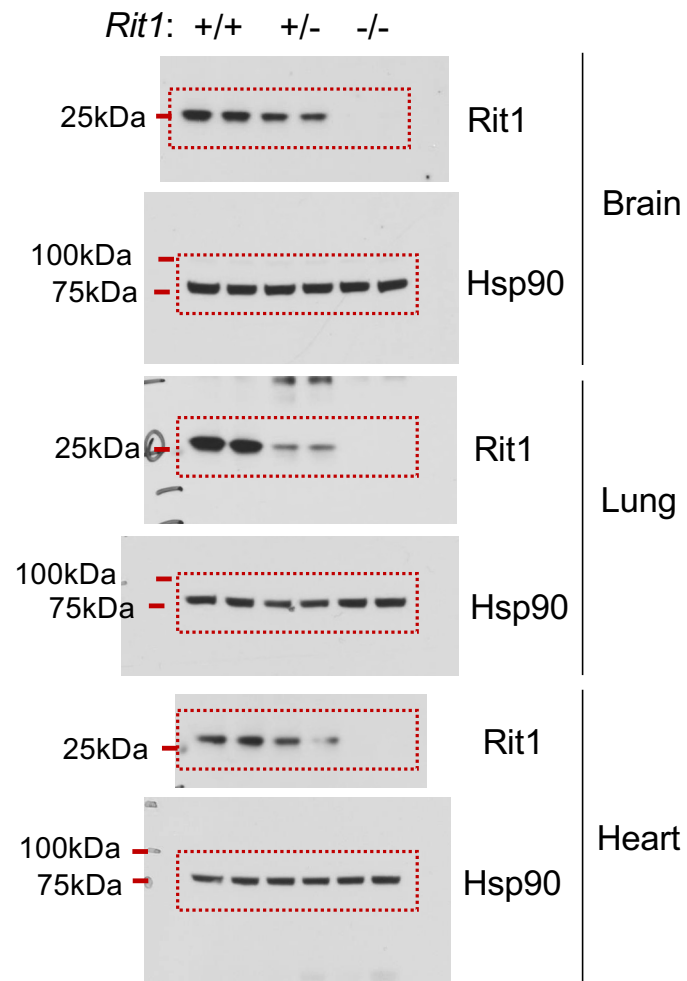

Supplement: Figure 4—source data 1. [file elife-76495-fig4-data1.zip › Figure 4 - source data/Figure 4 - source data 2.pdf]
