# Supplementary material for: Cross-species analysis of LZTR1 loss-of-function mutants demonstrates dependency to RIT1 orthologs
Source: eLife. 2022 Apr 25;11:e76495. doi: 10.7554/eLife.76495 (PMC9068208; doi:10.7554/eLife.76495)
Supplement: Figure 2—source data 1. [file elife-76495-fig2-data1.zip › Figure 2 - source data/Figure 2 - source data 1.pdf]

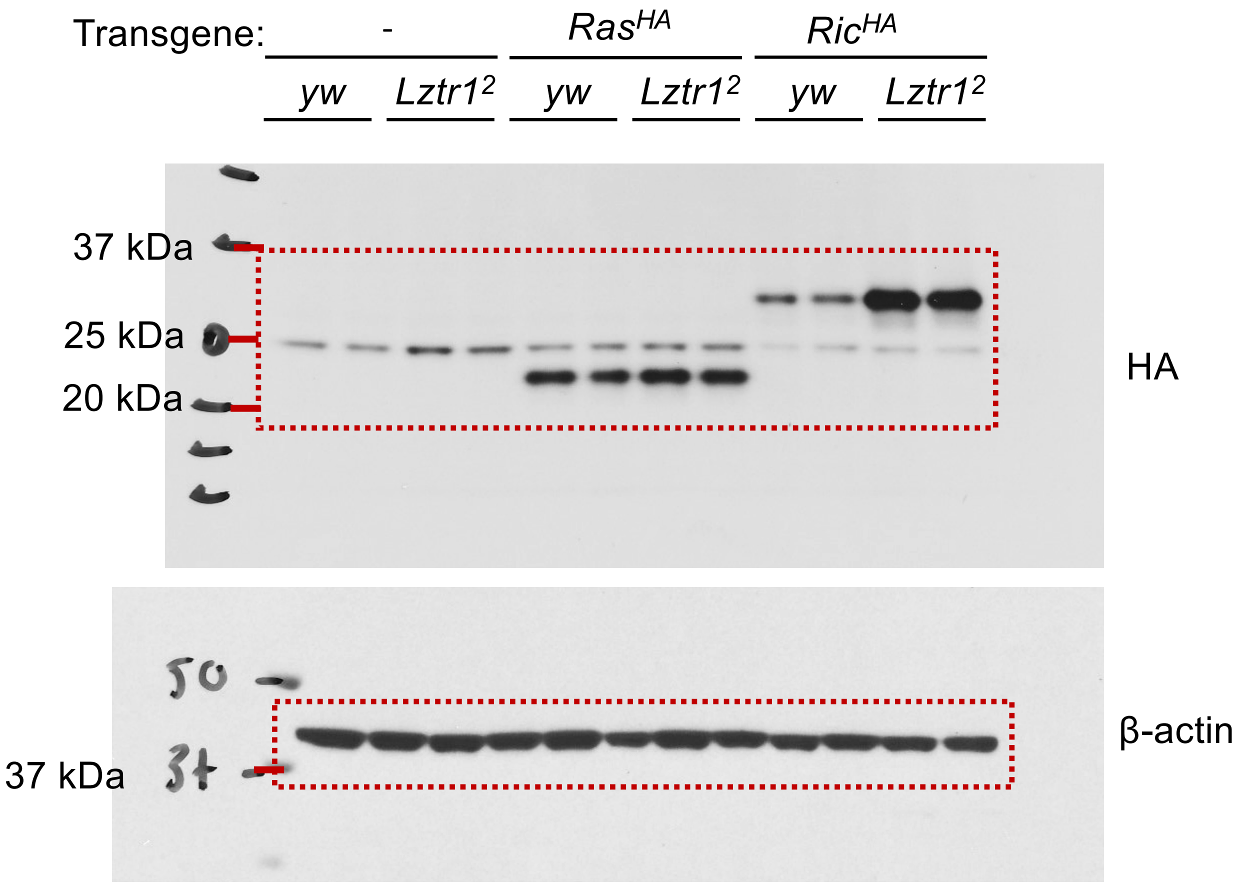
Transgene:
-
Ras^HA
Ric^HA
yw
Lztr1²
yw
Lztr1²
yw
Lztr1²
37 kDa
25 kDa
20 kDa
HA
50
37
37 kDa
β-actin
